# A century of Makerere and 22 years of academic publishing: African Health Sciences into a new gear

**DOI:** 10.4314/ahs.v22i2.1S

**Published:** 2022-08

**Authors:** James K Tumwine

Welcome to this special issue of *African Health Sciences* (AHS). Special, because it is a year of celebration. For Makerere: 100 years of remarkable existence and for *African Health Sciences* 22 years of unprecedented growth and development. How did it all begin?

Come August 2001, Prof. Nelson Sewankambo, then Dean of the Makerere University Medical School, assisted me to launch *African Health Sciences* amidst skepticism surrounding a “poor reading culture” etc.

Within 3 years, AHS was on MEDLINE/PUBMED, and 5 years later on WEB of Science. We have a modest impact factor of 1.18 this year, and last year we exceeded 2000 manuscripts per year managing to publish only 10% of what we get. We are very grateful to our partners through the African Journals Project (AJPP), for their unqualified material and professional support. We rely on volunteers 100%, and do not charge authors or readers - a unique form of Open Access policy. Our authors and readers love it. They do not need a credit card to reach us.

Now AHS must turn a new page: to become a catalyst in Makerere University's quest to become a truly research university. It is with that in mind that we have to look for innovative ways of involving Makerere University research community to join AHS as authors, editors, reviewers etc. In the same vein, we welcome the re-appointment of Prof. Nawangwe as Vice Chancellor, and know that we have an ally at the helm of this great institution.

## Editor's choice!

In this special issue, we bring you papers reflecting on capacity building for health care, research, and training,^1^,^2^ and research to policy initiatives in child health^3^, as well as infectious disease prevention.^4^ Collaboration in population based research^4^–^6^, as well as health service delivery^7^ completes this treatise on the core roles of a university such as Makerere.

A venture into high tech medicine^8^, PhD training^9^, and quick response to pandemics^10^–^13^ highlights Makerere's diverse roles. We give the readers a glimpse into surgery^14^, refugee health^15^ and eyecare^16^ as we conclude themes of this special issue.

As we begin a new century of education, research and service, we need to shift into a new gear of high quality research and publication. And *African Health Sciences* will be at the helm of that effort -catalyzing the publication of innovative high quality scientific papers relevant to Africa and the diaspora.
